# Machine Learning Algorithms Predict Successful Weaning From Mechanical Ventilation Before Intubation: Retrospective Analysis From the Medical Information Mart for Intensive Care IV Database

**DOI:** 10.2196/44763

**Published:** 2023-11-14

**Authors:** Jinchul Kim, Yun Kwan Kim, Hyeyeon Kim, Hyojung Jung, Soonjeong Koh, Yujeong Kim, Dukyong Yoon, Hahn Yi, Hyung-Jun Kim

**Affiliations:** 1 Division of Hematology-Oncology Department of Internal Medicine Inha University College of Medicine and Hospital Incheon Republic of Korea; 2 Department of the Technology Development Seers Technology Co, Ltd Seongnam Republic of Korea; 3 Crowdworks Co, Ltd Seoul Republic of Korea; 4 Healthcare Artificial Intelligence Team National Cancer Center Goyang Republic of Korea; 5 Department of Preventive Medicine Yonsei University Wonju College of Medicine Wonju Republic of Korea; 6 Department of Biomedical Systems Informatics Yonsei University College of Medicine Yongin Republic of Korea; 7 Asan Medical Center Asan Institute for Life Sciences Seoul Republic of Korea; 8 Division of Pulmonary and Critical Care Medicine Department of Internal Medicine Seoul National University Bundang Hospital Seongnam Republic of Korea; 9 Department of Internal Medicine Seoul National University College of Medicine Seoul Republic of Korea

**Keywords:** algorithms, clinical decision-making, intensive care units, noninvasive ventilation, organ dysfunction scores

## Abstract

**Background:**

The prediction of successful weaning from mechanical ventilation (MV) in advance of intubation can facilitate discussions regarding end-of-life care before unnecessary intubation.

**Objective:**

We aimed to develop a machine learning–based model that predicts successful weaning from ventilator support based on routine clinical and laboratory data taken before or immediately after intubation.

**Methods:**

We used the Medical Information Mart for Intensive Care IV database, which is an open-access database covering 524,740 admissions of 382,278 patients in Beth Israel Deaconess Medical Center, United States, from 2008 to 2019. We selected adult patients who underwent MV in the intensive care unit (ICU). Clinical and laboratory variables that are considered relevant to the prognosis of the patient in the ICU were selected. Data collected before or within 24 hours of intubation were used to develop machine learning models that predict the probability of successful weaning within 14 days of ventilator support. Developed models were integrated into an ensemble model. Performance metrics were calculated by 5-fold cross-validation for each model, and a permutation feature importance and Shapley additive explanations analysis was conducted to better understand the impacts of individual variables on outcome prediction.

**Results:**

Of the 23,242 patients, 19,025 (81.9%) patients were successfully weaned from MV within 14 days. Using the preselected 46 clinical and laboratory variables, the area under the receiver operating characteristic curve of CatBoost classifier, random forest classifier, and regularized logistic regression classifier models were 0.860 (95% CI 0.852-0.868), 0.855 (95% CI 0.848-0.863), and 0.823 (95% CI 0.813-0.832), respectively. Using the ensemble voting classifier using the 3 models above, the final model revealed the area under the receiver operating characteristic curve of 0.861 (95% CI 0.853-0.869), which was significantly better than that of Simplified Acute Physiology Score II (0.749, 95% CI 0.742-0.756) and Sequential Organ Failure Assessment (0.588, 95% CI 0.566-0.609). The top features included lactate and anion gap. The model’s performance achieved a plateau with approximately the top 21 variables.

**Conclusions:**

We developed machine learning algorithms that can predict successful weaning from MV in advance to intubation in the ICU. Our models can aid the appropriate management for patients who hesitate to decide on ventilator support or meaningless end-of-life care.

## Introduction

Acute respiratory failure can be caused by various conditions, including pulmonary disease, cardiovascular disease, and neuromuscular disorder, or required respiratory support after major surgery [1]. Although invasive mechanical ventilation (MV) is a life-maintaining intervention used to assist or replace spontaneous respiration in patients with acute respiratory failure, the procedure is associated with a risk of severe complications such as ventilator-associated pneumonia, pulmonary edema, and acute respiratory distress syndrome [2].

Inevitably, a proportion of patients will be unable to recover rapidly from ventilator support, mandating the use of MV for an extended period. The duration of MV is linearly associated with poor outcomes, and the number of days of ventilation support directly correlates with daily incremental costs and unexpected medical conditions such as thromboembolic events and posttraumatic stress disorder [3]. Prolonged MV unavoidably accompanies tracheostomy and life-maintaining care, which is not usually desired by patients [4,5]. Such a decision is usually made within 14 days of MV [6]. Tracheostomy has its advantages in a lower frequency of laryngeal ulcers, less airway resistance, and ease of management [4,5]. However, prolonged MV and consequent tracheostomy are unlikely to benefit chronically ill patients with an expected dismal prognosis. It should only be performed if it aligns with the patient’s goals and preferences. The possibility of undergoing a tracheostomy can be a reason for hesitancy to intubate older or chronically ill patients.

For these reasons, successful early predictions of whether a patient will undergo prolonged MV can support clinical decision-making in many clinical aspects. Several risk factors, including underlying comorbidities, the site of intubation, laboratory or blood gas results, functional parameters, and critical care scoring systems, have been identified for successful weaning from MV [7]. However, as the predictive power using a single or a few variables was insufficient, there have been efforts to create a predictive model by assigning weights to each relevant variable.

Machine learning could potentially be a breakthrough in this type of prediction, where various factors are involved in a complex manner. Several studies have used machine learning techniques to predict successful extubation using a combination of multiple variables [8]. However, the models proposed to predict successful weaning from MV did not reflect various clinical situations requiring prediction before initiating MV, because most models collected variables and predicted outcomes at the time of MV progress, not before intubation. Other previous models have been suggested to anticipate prolonged MV or tracheostomy [9]. However, they were either short-term predictive models or unrealistic models that only predicted whether patients would receive prolonged MV but excluded patients who died before MV day 14. Given this background, we aimed to develop a thorough machine learning model that can predict the possibility of successful weaning from MV within 14 days after intubation, before undergoing intubation.

## Methods

### Data Source

Data on patients requiring MV were obtained from the Medical Information Mart for Intensive Care IV (MIMIC-IV) version 1.0 database. MIMIC-IV is a well-known, large-scale, single-center (Beth Israel Deaconess Medical Center), and open-access database covering 524,740 admissions of 382,278 patients to the center from 2008 to 2019 [10]. The relevant records include demographic data; International Classification of Diseases, Ninth Revision, Clinical Modification codes; hourly vital signs and input or output; laboratory tests and microbiological culture results; imaging data, treatment procedures; medication administration; and survival data. The database also provides multiple severity-of-illness scores generated from physiologic and laboratory variables on the first day of each intensive care unit (ICU) admission. MIMIC-IV has several advantages over its previous version, MIMIC-III. The composing data are relatively homogenous, because MIMIC-IV contains data entirely sourced from the clinical information system MetaVision (iMDSoft); the information of “procedure events,” one of the primary sources of clinical information in ICU, is entirely present; and a substantial number of patients is included.

### Selection of Participants

For meticulous patient selection, patients with “Intubation” and “Invasive ventilation” codes appearing at least once in the “procedure event” or “chart event” files were selected. Additionally, patients with “Ventilator type” and “Ventilator mode” codes appearing 5 times or more within 24 hours after the first code were also included (Figure S1 in Multimedia Appendix 1). The exclusion criteria were as follows: (1) aged <18 or >100 years, (2) previous tracheostomy, and (3) missing Sequential Organ Failure Assessment (SOFA) score and Simplified Acute Physiology Score II (SAPS II).

### Data Collection and Outcome Definition

We collected clinical and laboratory variables recorded before and closest to the initiation of MV. For patients who did not have the values before intubation, the nearest value was obtained within 24 hours of intubation. To minimize the impact of intubation on each variable, we selected variables that are less likely to change dramatically after intubation. Clinical and laboratory variables considered relevant to patient prognosis in the ICU were selected by 2 clinicians (JK and HJK) from the list of variables included in the MIMIC-IV database. Any discrepancy was resolved by group discussion. They were as follows: age, sex, height, weight, Glasgow Coma Scale (eye), Glasgow Coma Scale (motor), hemoglobin, pH, lactate, albumin, anion gap, total bilirubin, bicarbonate, blood urea nitrogen, creatinine, platelet count, prothrombin time, neutrophil or lymphocyte ratio, sodium, potassium, white blood cell count, body temperature, type of admission (medical, scheduled surgical, and unscheduled surgical), type of insurance (public and other), primary language (English and other), marital status (couple and single), race and ethnicity (Asian, Black, Hispanic, White, and others), type of ICU (medical, surgery, and others), and underlying comorbidities (myocardial infarction, congestive heart failure, peripheral vascular disease, cerebrovascular disease, dementia, chronic pulmonary disease, rheumatic disease, peptic ulcer disease, mild liver disease, diabetes without complication, diabetes with complication, paraplegia, renal disease, malignancy, severe liver disease, metastatic solid tumor, and acquired immune deficiency syndrome).

The missing values were imputed using multiple imputations by chained equations for continuous variables and the k-nearest neighbor method for categorical variables. The missing rates are depicted in Figure S2 in Multimedia Appendix 1. As a comparator, 2 severity-of-illness scores, that is, the SOFA and SAPS II scores, were calculated using the codes from Google’s BigQuery database [11]. The primary outcome was successful weaning within 14 days of intubation, defined as documented MV discontinuation without death.

### Model Development

The development and validation of our model were performed according to the “Guidelines for Developing and Reporting Machine Learning Predictive Models in Biomedical Research” [12]. The checklist is available in Table S1 in Multimedia Appendix 1. Several machine learning algorithms were used to develop predictive models, such as regularized logistic regression classifier (RLRC) [13], random forest classifier (RFC) [14], CatBoost classifier (CBC) [15], and voting classifier (VC) ensembles [16]. Since the data set was imbalanced, we used an algorithm-level approach to handle the imbalance. Specifically, we used Cohen κ maximizing threshold of the threshold-moving approach, cost-sensitive learning, and ensemble method of VC to reduce bias or variance and improve the stability of machine learning algorithms [17-19].

We used the 5-fold cross-validation method with a fixed random seed to achieve reproducibility. The 5-fold cross-validation divides the data into 5 equal partitions, training the model on 4 and testing it on the remaining 1. This process is repeated 5 times, with each partition used once as the test set. The performance metric is the average performance across all 5 iterations. This method is a way to demonstrate a model’s robustness in the absence of external data and avoids the risk of model overfitting. Mean with 95% CIs of the area under the receiver operating characteristic curve (AUROC), the area under the precision-recall curve (AUPRC), Cohen κ, and *F*_1_-score of the models were calculated using the 5-fold cross-validation. The AUPRC and AUROC were used to verify the effectiveness of our proposed model and compare it with other models. AUROC can be used as a diagnostic test to discriminate between actual positives and negatives. However, AUPRC is used as an alternative to AUROC for tasks with highly skewed class distribution [20,21]. Although AUROC can evaluate classifiers when there is a class imbalance, it can present an overly optimistic view of performance if there is a large skew in the class distribution [22]. Thus, we showed both AUROC and AUPRC to prove the superiority of our proposed model to other models.

Cohen κ is a metric for evaluating the classification algorithm’s consistency based on its predictions [22]. *F*_1_-score is also used as an evaluation and comparison metric. *F*_1_-score is the measure of the weighted average of 2 evaluation metrics of precision and sensitivity. Therefore, we chose the *F*_1_-score to obtain harmonic means between precision and sensitivity. Likewise, the performance parameters for the SOFA and SAPS II scores were calculated, and the AUROCs of each model were compared using the DeLong test [23].

The VC model’s confusion matrix was presented using Cohen κ maximizing threshold value. To determine the model’s threshold, we performed a manual visual inspection with a graph of the threshold versus Cohen κ [17]. After exploring the threshold at which Cohen κ value can be maximized, we presented this result in the confusion matrix by adjusting true positives, true negatives, false positives, and false negatives using the threshold at which this value can be maximized.

To determine the optimal hyperparameter setting, the *GridSearchCV* library (version 0.22) was used to search multiple optimal parameter values to fit estimators automatically. We drew calibration plots for each algorithm (Figure S3 in Multimedia Appendix 1) [24,25]. Finally, to better understand how individual variables impact the outcome prediction, a permutation feature importance and Shapley additive explanations (SHAP) analysis on the best-performing model was conducted [14,26]. For model development and validation, Python (version 3.6.9; Python Software Foundation) and its packages such as *NumPy* (version 1.19.5) [27], *pandas* (version 1.1.5) [28], *scikit-learn* (version 0.23.2) [29], *Matplotlib* (version 3.3.4) [30], *seaborn* (version 0.11.2) [31], *rpy2* (version 3.4.5) [32], *SciPy* (version 1.5.4; Enthought) [33], and *SHAP* (version 0.41.0) [26,34], as well as R (version 3.4.4; R Core Team) [35] and its package *pROC* (version 1.18.0) [23], were used [36]. The codes used in this study are made available at GitHub [37] for noncommercial use.

### Other Statistical Considerations

To compare the baseline characteristics, categorical variables were presented as total numbers (percentages) and compared using Fisher exact test. Continuous variables were presented as means (SD) and compared using the Wilcoxon rank-sum test. All statistical analyses in this study were performed using Google BigQuery, Python (version 3.6.9), and R (version 3.4.4), and *P*<.05 were considered statistically significant.

### Ethical Considerations

The research resource, which includes the collection of patient information, was assessed by the Institutional Review Board at Beth Israel Deaconess Medical Center. They provided a waiver of informed consent and endorsed the data-sharing initiative. Furthermore, the study received approval from the Institutional Review Board of Seoul National University Bundang Hospital (B-2201-733-002).

## Results

### Baseline Characteristics

**Figure 1:**
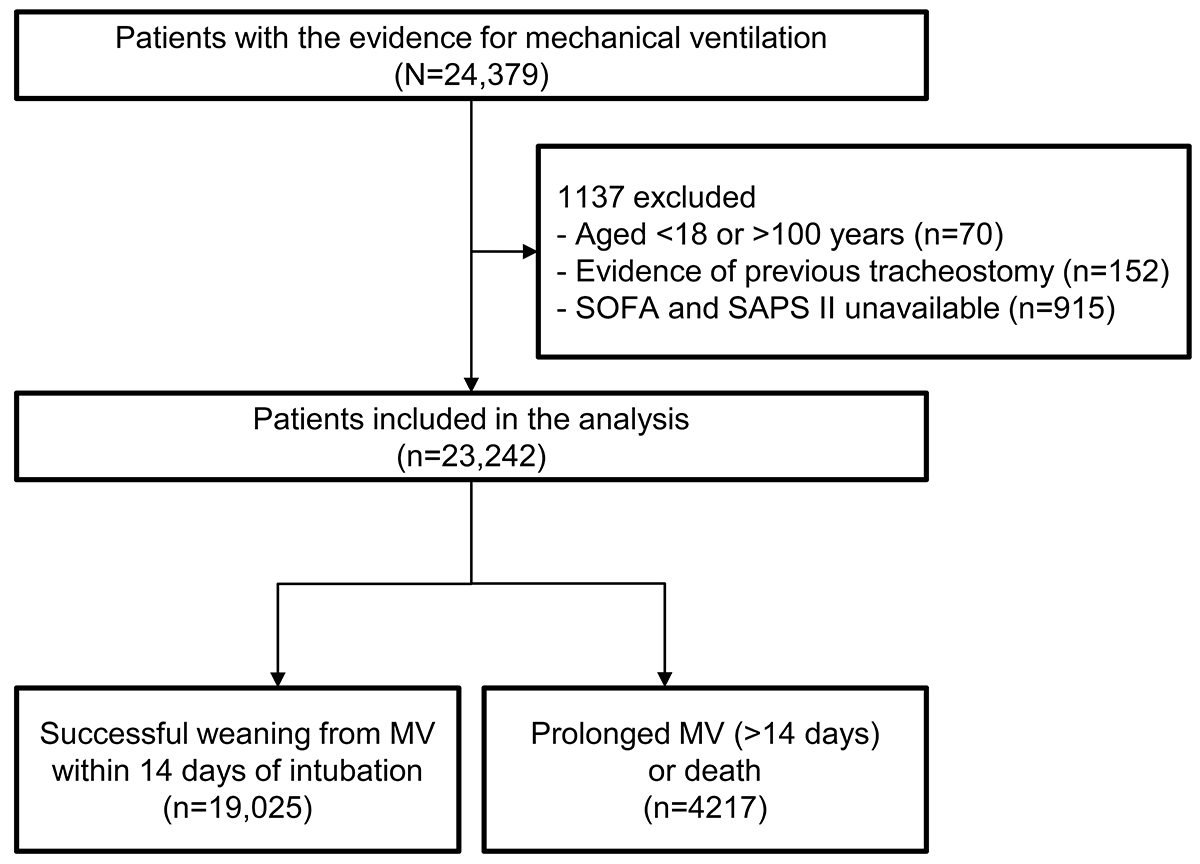
Flowchart of the patient selection process. Patients with evidence of endotracheal intubation were identified in the Medical Information Mart for Intensive Care IV database. After careful selection, patients were divided into 2 groups according to whether they had been successfully weaned from MV within 14 days of intubation or not. MV: mechanical ventilation; SAPS II: Simplified Acute Physiology Score II; SOFA: Sequential Organ Failure Assessment.

Out of the 24,379 patients screened, 23,242 were included in the analysis, and 19,025 (81.9%) successfully weaned from MV within 14 days (Figure 1). For those who successfully weaned from MV, the duration of MV (mean 1.7, SD 2.7 vs mean 8.1, SD 10.6 d; *P*<.001), age (mean 63.7, SD 15.9 vs mean 67.8, SD 15.8 y; *P*<.001), blood urea nitrogen (mean 22.4, SD 17.2 vs mean 33.1, SD 25.4 mg/dL; *P*<.001), creatinine (mean 1.2, SD 1.2 vs mean 1.7, SD 1.5 mg/dL; *P*<.001), anion gap (mean 13.7, SD 4.0 vs mean 17.3, SD 5.8 mEq/L; *P*<.001), SAPS II (mean 37.5, SD 13.3 vs mean 51.6, SD 16.7; *P*<.001), and SOFA score (mean 2.7, SD 2.5 vs mean 3.9, SD 3.5; *P*<.001) were lower, whereas pH (mean 7.4, SD 0.1 vs mean 7.3, SD 0.1; *P*<.001) and bicarbonate level (mean 23.4, SD 4.2 vs mean 21.1, SD 5.9 mEq/L; *P*<.001) were higher than the other group of patients (Table 1). The proportions of patients with public insurance (56.5% vs 48.7%; *P*<.001), single marital status (60.1% vs 51.2%; *P*<.001), and admission to a medical care unit (36.3% vs 19.1%; *P*<.001) were lower for those who had been weaned from MV within 14 days. Underlying comorbidities were also less common in these patients (Table S2 in Multimedia Appendix 1).

**Table 1:** Baseline characteristics of patients in the intensive care unit according to successful weaning from mechanical ventilation within 14 days.

Variables	All patients (N=23,242)	Successful weaning (n=19,025)	Prolonged mechanical ventilation or mortality (n=4217)	*P* value
Age (y), mean (SD)	64.4 (15.9)	63.7 (15.9)	67.8 (15.8)	<.001
Male sex, n (%)	14,274 (61.4)	11,935 (62.7)	2339 (55.5)	<.001
Height (cm), mean (SD)	169.7 (10.8)	170 (10.8)	168.7 (10.9)	<.001
Weight (kg), mean (SD)	83.3 (23.2)	83.8 (22.7)	81.0 (25.4)	<.001
Body temperature (°C), mean (SD)	36.6 (1.3)	36.7 (1.2)	36.5 (1.6)	<.001
WBC^a^ count (per 10^9^/L), mean (SD)	13.0 (7.5)	12.7 (6.9)	14.3 (9.8)	<.001
NL^b^ ratio, mean (SD)	9.1 (10.9)	8.3 (9.3)	12.3 (15.7)	<.001
Hemoglobin (g/dL), mean (SD)	10.4 (2.1)	10.4 (2.1)	10.6 (2.4)	<.001
Platelet count (per 10^9^/L), mean (SD)	190.4 (100.9)	188.9 (97.1)	197.4 (116.2)	<.001
BUN^c^ (mg/dL), mean (SD)	24.4 (19.4)	22.4 (17.2)	33.1 (25.4)	<.001
Creatinine (mg/dL), mean (SD)	1.3 (1.3)	1.2 (1.2)	1.7 (1.5)	<.001
Albumin (g/dL), mean (SD)	3.2 (0.4)	3.2 (0.4)	3.1 (0.5)	<.001
Total bilirubin (mg/dL), mean (SD)	1.3 (3.1)	1.1 (2.4)	2.2 (5.2)	<.001
Prothrombin time (INR^d^), mean (SD)	1.5 (0.7)	1.4 (0.6)	1.7 (1.1)	<.001
pH, mean (SD)	7.3 (0.1)	7.4 (0.1)	7.3 (0.1)	<.001
Sodium (mEq/L), mean (SD)	138.9 (4.6)	138.9 (4.3)	138.9 (5.9)	.900
Potassium (mEq/L), mean (SD)	4.2 (0.7)	4.2 (0.7)	4.3 (0.8)	<.001
Lactate (mmol/L), mean (SD)	2.6 1.9)	2.4 (1.5)	3.7 (3.1)	<.001
Bicarbonate (mEq/L), mean (SD)	23.0 (4.7)	23.4 (4.2)	21.1 (5.9)	<.001
Anion gap (mEq/L), mean (SD)	14.4 (4.6)	13.7 (4.0)	17.3 (5.8)	<.001
SOFA^e^, mean (SD)	2.9 (2.8)	2.7 (2.5)	3.9 (3.5)	<.001
SAPS II^f^, mean (SD)	40.0 (15.0)	37.5 (13.3)	51.6 (16.7)	<.001
Duration of MV^g^ (days), mean (SD)	2.8 (5.7)	1.7 (2.7)	8.1 (10.6)	<.001

^a^WBC: white blood cell.

^b^NL: neutrophil or lymphocyte.

^c^BUN: blood urea nitrogen.

^d^INR: international normalized ratio.

^e^SOFA: Sequential Organ Failure Assessment.

^f^SAPS II: Simplified Acute Physiology Score II.

^g^MV: mechanical ventilator.

### Development of the Prediction Models

**Figure 2:**
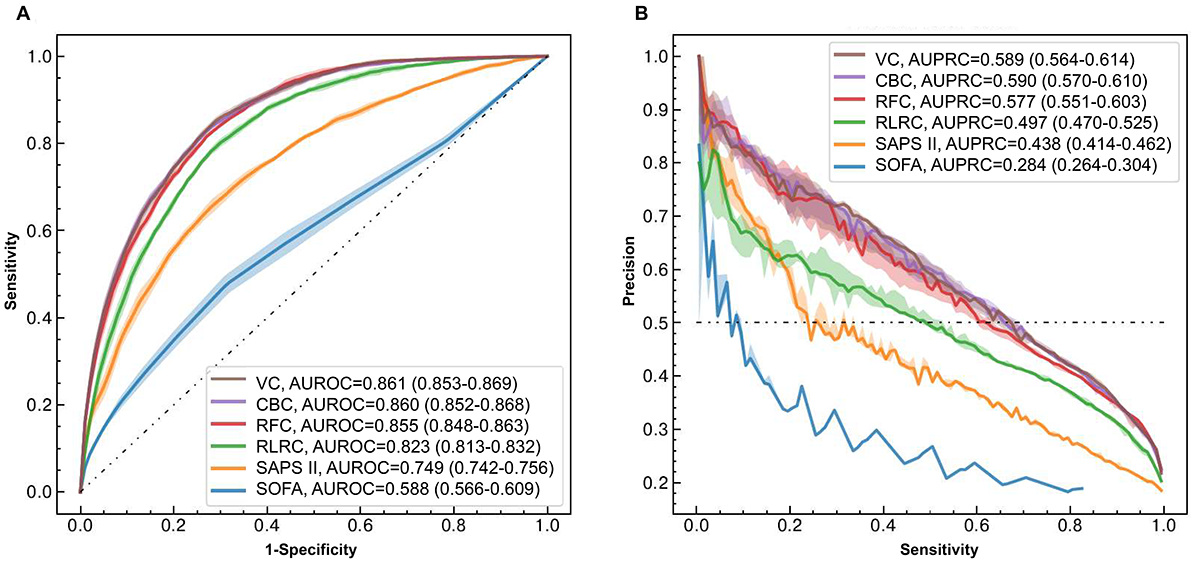
(A) Receiver operating characteristic curves and (B) precision-recall curves of the developed machine learning models to predict successful weaning from mechanical ventilation within 14 days of intubation. The area under receiver operating characteristics curves (AUROCs) and area under precision-recall curves (AUPRCs) and their 95% CIs for all 6 models are shown in the legends. For comparability, the random classifier is indicated by a black dashed-dotted line. CBC: CatBoost classifier; RFC: random forest classifier; RLRC: regularized logistic regression classifier; SAPS II: Simplified Acute Physiology Score II; SOFA: Sequential Organ Failure Assessment; VC: voting classifier.

The AUROC and AUPRC values resulting from 5-fold cross-validation are shown in Figure 2. The AUROC values (Figure 2A) of the VC, CBC, RFC, and RLRC models for the prediction of successful weaning were 0.861 (95% CI 0.853-0.869), 0.860 (95% CI 0.852-0.868), 0.855 (95% CI 0.848-0.863), and 0.823 (95% CI 0.813-0.832), respectively. The 2 conventional scoring models showed AUROC values of 0.749 (95% CI 0.742-0.756) for SAPS II and 0.588 (95% CI 0.566-0.609) for the SOFA score. Figure 2B presents the positive predictive value against sensitivity, with AUPRC values of 0.589 (95% CI 0.564-0.614), 0.590 (95% CI 0.570-0.610), 0.577 (95% CI 0.551-0.603), and 0.497 (95% CI 0.470-0.525), respectively, in each machine learning model. Detailed descriptions of various performance metrics are presented in Table 2. We also generated a confusion matrix for the cross-validation of the VC model (Figure S4 in Multimedia Appendix 1).

**Table 2:** Performance metrics of the developed machine learning models, along with SOFA^a^ score and SAPS II^b^,^c^.

Model	AUROC^d^, mean (95% CI)	AUPRC^e^, mean (95% CI)	Cohen κ, mean (95% CI)	*F*_1_-score, mean (95% CI)
VC^f^	0.861 (0.853-0.869)^g,h^	0.589 (0.564-0.614)	0.413 (0.404-0.421)	0.554 (0.550-0.558)
CBC^i^	0.860 (0.852-0.868)^g,h^	0.590 (0.570-0.610)	0.400 (0.383-0.417)	0.546 (0.536-0.557)
RFC^j^	0.855 (0.848-0.863)^g,h^	0.577 (0.551-0.603)	0.392 (0.380-0.404)	0.540 (0.534-0.547)
RLRC^k^	0.823 (0.813-0.832)^g,h^	0.497 (0.570-0.525)	0.359 (0.348-0.369)	0.515 (0.509-0.521)
SAPS II	0.749 (0.742-0.756)^g^	0.438 (0.414-0.462)	0.280 (0.263-0.297)	0.451 (0.440-0.462)
SOFA	0.588 (0.566-0.609)^h^	0.284 (0.264-0.304)	0.121 (0.096-0.147)	0.330 (0.311-0.349)

^a^SOFA: Sequential Organ Failure Assessment.

^b^SAPS II: Simplified Acute Physiology Score II.

^c^Values were calculated from 5-fold cross-validation. Hypothesis tests were conducted to determine whether the AUROC values of the models using machine learning algorithms were equal to those of conventional scores.

^d^AUROC: area under the receiver operating characteristics curve.

^e^AUPRC: area under the precision-recall curve.

^f^VC: voting classifier.

^g^*P*<.001 compared to SOFA score.

^h^*P*<.001 compared to SAPS II.

^i^CBC: CatBoost classifier.

^j^RFC: random forest classifier.

^k^RLRC: regularized logistic regression classifier.

### Feature Importance of Each Variable

**Figure 3:**
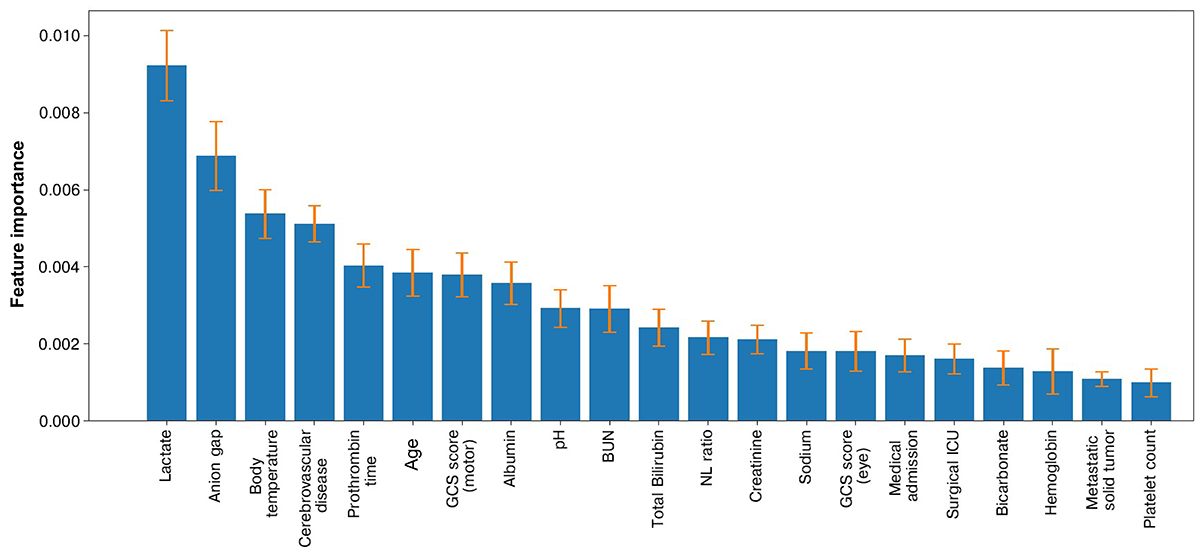
Important explanatory variables for the ensemble voting classifier model. The means and SD of the top 21 important explanatory variables for the voting classifier model using 5-fold cross-validation and test set permutation are indicated by bars and error bars, respectively. BUN: blood urea nitrogen; GCS: Glasgow Coma Scale; ICU: intensive care unit; NL: neutrophil-to-lymphocyte; WBC: white blood cell.

**Figure 4:**
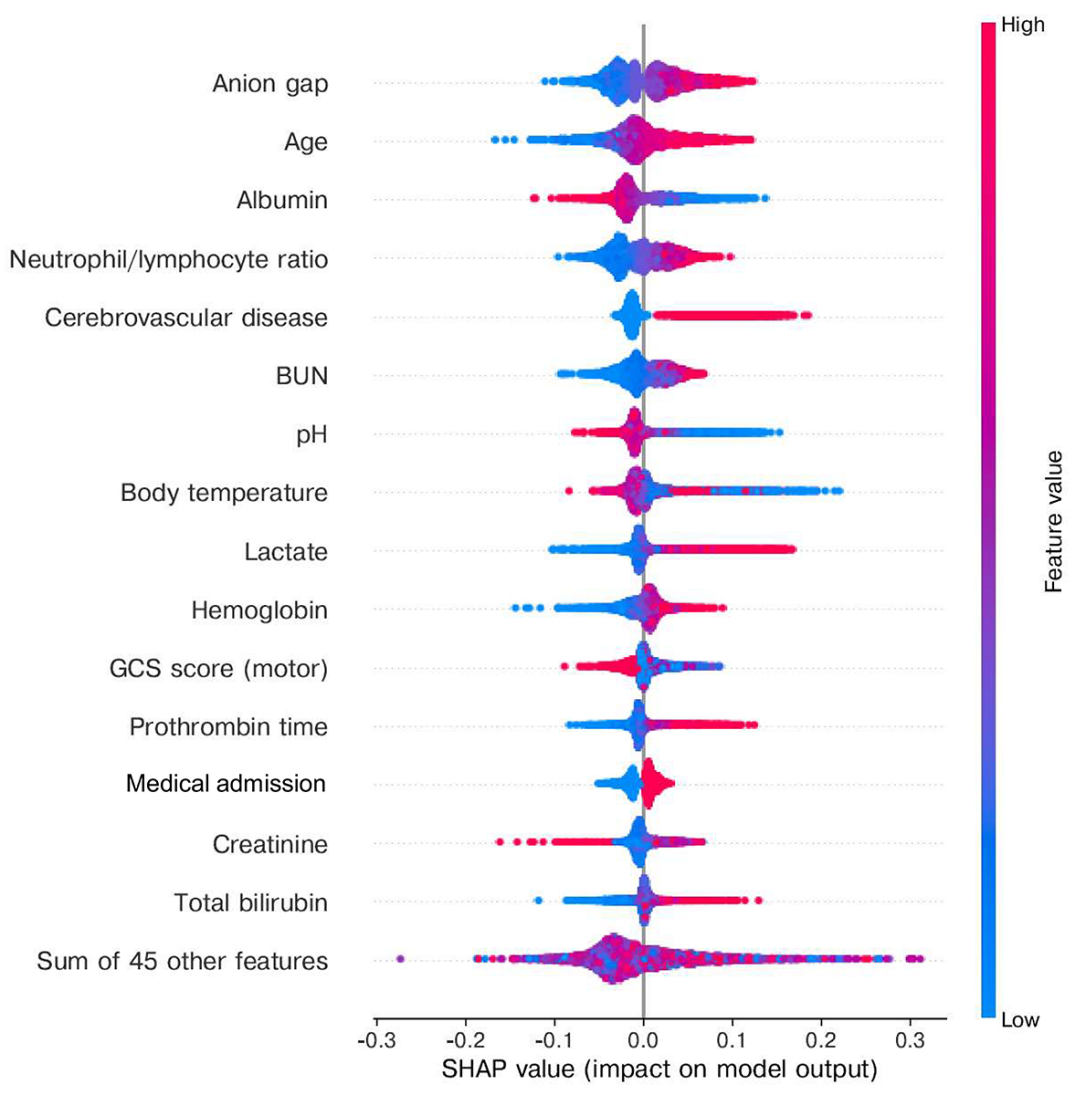
The Beeswarm summary plot in the SHAP package with a randomly selected test set among a 5-fold cross-validation. This figure indicates which explanatory variables have the most significant influence on the model’s predicted value and the tendency of the model’s outcome probability as a function of the original value of an explanatory variable simultaneously. Each colored dot represents one variable value for a patient, for which larger values are presented in red and smaller values in blue. The SHAP value, a simplified and computer-friendly Shapley value, is shown on the horizontal axis and is a surrogate of the contribution of a variable value to the model output. BUN: blood urea nitrogen; GCS: Glasgow Coma Scale; SHAP: Shapley additive explanations.

We chose the VC model as our representative model. The included variables in the model were ranked according to their information gain, and the top 3 features were lactate concentration, anion gap, and body temperature (Figure 3). To better understand the direction of influence each feature has on this model, the SHAP algorithm was implemented for this model to explain for each feature the magnitude and direction of its impact on the outcome prediction. The top risk features included anion gap, age, presence of cerebrovascular disease, and blood urea nitrogen concentration. Specifically, a higher value or the presence of a variable indicates a higher chance of failure to wean from MV within 14 days (Figure 4).

### Change of Model Performance With Variables

**Figure 5:**
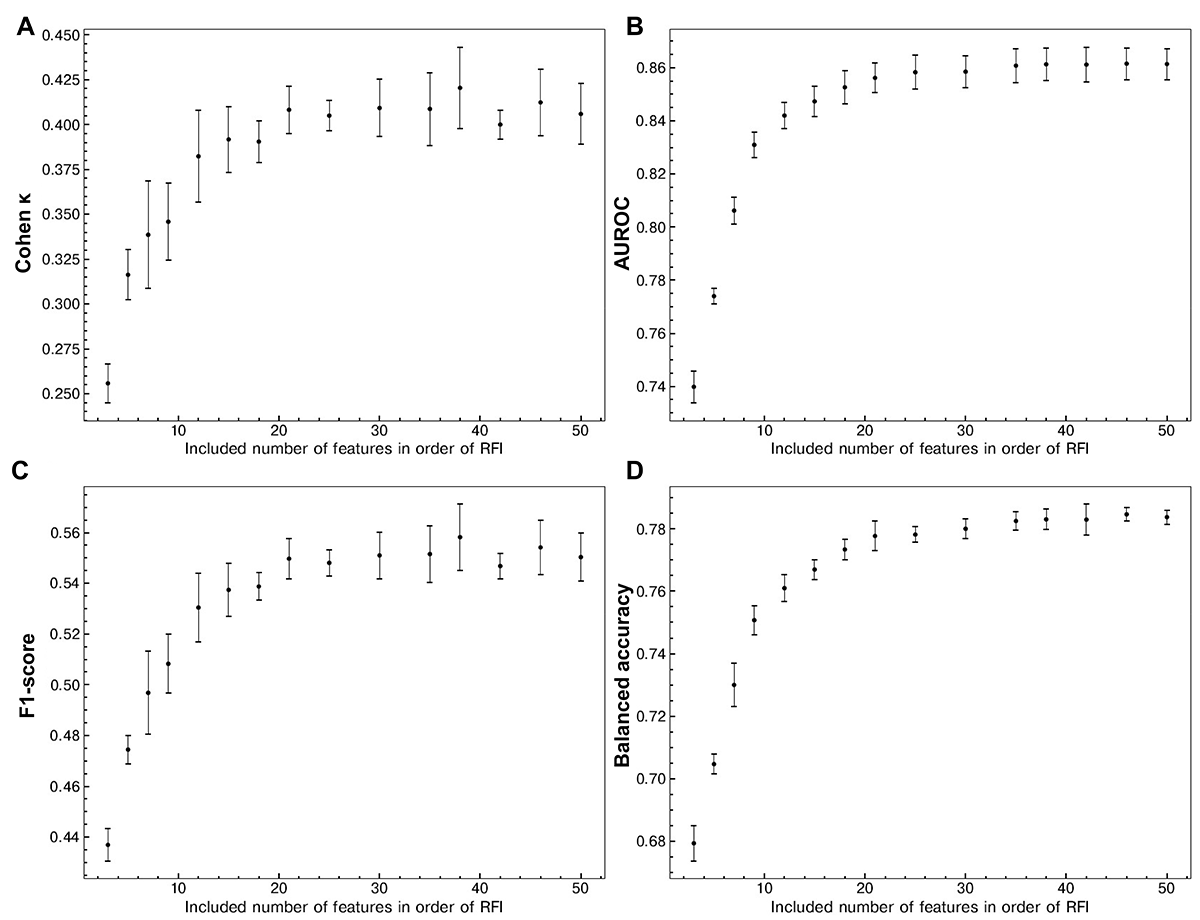
Performance metrics of the ensemble voting classifier model according to the addition of explanatory variables. (A) Cohen κ, (B) AUROC, (C) *F*_1_-score, and (D) balanced accuracy of the ensemble voting classifier are shown as the number of explanatory variables in the training sets in descending order of feature importance coefficients. Mean (dot) and SD (error bar) were computed from the data sets by 5-fold cross-validation. The model reached its plateau performance in all 4 metrics with approximately 21 variables. AUROC: area under the receiver operating characteristics curve; RFI: relative feature importance.

We assessed the performance metrics (Cohen κ, AUROC, *F*_1_-score, and balanced accuracy) of the VC model according to the number of features included (Figure 5). Each metric’s performance was calculated as the variables with the highest feature importance were sequentially added. The model reached its plateau performance in all 4 metrics with approximately 21 variables.

## Discussion

### Principal Findings and Clinical Implications

Within the context of the ICU, physicians often rely on clinical presentations to gauge the likelihood of a patient's successful weaning from MV prior to endotracheal intubation. Translating these clinical intuitions into quantifiable metrics, however, presents a challenge. To address this, our study meticulously developed and validated machine learning–based models designed to predict successful weaning from MV either before or immediately postintubation in critically ill patients on ventilator support. Notably, our model's predictive accuracy surpassed that of traditional prognostic scoring systems commonly used for ICU patients.

The practical use of our model is underscored by its ability to harness readily available data from electronic health records, including vital signs, foundational laboratory results, and patient medical histories. This facilitates the identification of patients at an elevated risk of extended MV reliance. The advantages of using our model are multifaceted. Prompt identification of high-risk patients allows for the timely initiation of therapeutic interventions, potentially curtailing the duration of MV. Such early identification ensures a judicious allocation of resources, optimizing the use of specialized equipment and expert personnel, leading to enhanced patient outcomes and cost-effectiveness. Additionally, it paves the way for proactive discharge planning, bolstering patient satisfaction, and alleviating pressures on the health care infrastructure.

### Comparison With Prior Work

This is the first study to develop a prognostic model that predicts relatively long-term outcomes (14 days) based on variables within a day of intubation. The model characteristics render it clinically pragmatic and facilitate improved discussions about end-of-life care or prolonged MV with a tracheostomy. Previous efforts to predict the prognosis of patients undergoing MV in ICUs have shown several drawbacks. Clark et al [9,38] suggested a model consisting of clinical variables (intubation in the ICU, tachycardia, renal dysfunction, acidemia, elevated creatinine, and decreased HCO_3_^–^ concentration) to identify individuals who may need prolonged MV at the time of intubation. However, their model was derived from and validated in relatively small patient populations (99 and 225 patients, respectively), and the AUROC value for prolonged MV prediction was about 0.75. Moreover, patients who died within 2 weeks of starting MV were excluded from the analysis, leading to a selection bias. Several other studies exist but only predicted mortality [39] and short-term outcomes [40], or they did not consider death in model building [41].

Numerous clinical factors have been proposed in predictive models for patients with MV. The I-TRACH (Intubation in the ICU, Tachycardia, Renal Dysfunction, Acidemia, Creatinine, and Decreased HCO_3_) model previously extracted tachycardia, renal dysfunction, acidemia, and a decreased HCO_3_^–^ concentration as the main variables for constructing a scoring system [38]. In another study that reported the prediction of 30-day mortality, the essential features in the models were Acute Physiology and Chronic Health Evaluation II score, Charlson Comorbidity Index, use of norepinephrine, and base excess [39]. In our study, lactate level and anion gap were the 2 most important predictors in the final VC model. Per the findings of this study, several prior reports have emphasized the prognostic importance of lactate level and anion gap. The efficacy of early lactate-guided therapy in ICU patients has been reported [42]. The anion gap, a surrogate for levels of unmeasured anions, has been reported in a meta-analysis as an indicator of mortality in critically ill patients [43].

### Strengths

We proposed the VC ensemble comprising RLRC, RFC, and CBC as our representative model. Historically, logistic and linear regression models have been used for prognosis tasks in clinical decisions concerning ICU-admitted patients [44]. Still, they are not fit for predictor variables with skewed distributions and tend to overfit. To avoid these shortcomings, more complex modeling approaches have been proposed [45]. First, RLRC does not rely on multivariate normality and equal within-group covariance matrices, but predictions require large-scale sample data for stable outcomes [13,46]. Second, RFC works well on data with several input variables and improves its classification accuracy because it keeps bias low and reduces variance. Still, the interpretation is complex, and evaluation is slow [14]. Third, CBC requires lower computational costs but shows better accuracy than other tree-based models and support vector machines [15,47]. The 3 models (RLRC, RFC, and CBC) showed a tradeoff between precision and recall [48]; therefore, the use of the VC ensemble method improves performance by reducing the variance component of prediction errors made by the contributing models [16].

Apart from the thorough development of the ensemble model, our study has its strength due to the relatively high number of included patients (n=23,242) provided by the MIMIC-IV database, which is a well-established open database derived from an ICU in the United States. This is the first study to establish a machine learning model to predict the weaning probability based on MIMIC-IV data. Moreover, precise search terms such as “intubation/invasive ventilation” and “ventilator type/mode” were used to establish a more stringent patient selection and outcome definition. Such criteria can provide an example for the selection processes of intubated patients for future studies using the MIMIC-IV database.

### Limitations

Despite our meaningful findings, there are some inherent limitations to our study. First, some imbalance in patient numbers was noted between those with and without successful weaning from MV (81.9% vs 18.1%, respectively). Therefore, we used an algorithm-level approach to handle the imbalance and presented various performance metrics such as AUPRC and Cohen κ. Second, some variables had a considerable proportion of missing data or were collected after intubation. Although small, 915 patients with missing SOFA and SAPS II scores were also excluded from our analysis, leading to concerns of selection bias (Table S3 in Multimedia Appendix 1). However, the presence of missing data reflects our real-world medical practice, and we used variables not likely to change dramatically according to intubation (eg, body temperature, blood urea nitrogen, and underlying diseases). Third, our model lacks external validity due to its innate nature as a single-center study. However, our model is grounded on universally recognized variables, including vital signs, underlying comorbidities, and laboratory findings. The open nature of our data set, combined with the web-based availability of our code, simplifies validation efforts for other researchers. Future studies for external validation can strengthen our models.

### Conclusions

In conclusion, we developed and validated a VC ensemble machine learning model that can effectively predict successful weaning from MV within 14 days before or immediately after intubation. Our study indicates that machine learning algorithms may facilitate clinical decision-making, such as identifying patients more likely to benefit from MV before or immediately after endotracheal intubation. This information can relieve the burden and aid doctors in suggesting appropriate management for patients at risk of endotracheal intubation in the ICU, notably for those who hesitate to decide on ventilator support or meaningless end-of-life care due to advanced age or the presence of several comorbidities.

## Appendix

Multimedia Appendix 1 Supplementary tables and figures on guidelines and additional data not presented in the main text.

## Abbreviations

AUPRC: area under the precision-recall curve
AUROC: area under the receiver operating characteristic curve
CBC: CatBoost classifier
I-TRACH: Intubation in the Intensive Care Unit, Tachycardia, Renal Dysfunction, Acidemia, Creatinine, and Decreased HCO3
ICU: intensive care unit
MIMIV-IV: Medical Information Mart for Intensive Care IV
MV: mechanical ventilation
RFC: random forest classifier
RLRC: regularized logistic regression classifier
SAPS II: Simplified Acute Physiology Score II
SHAP: Shapley additive explanations
SOFA: Sequential Organ Failure Assessment
VC: voting classifier
