# Supplementary material for: Machine Learning Algorithms Predict Successful Weaning From Mechanical Ventilation Before Intubation: Retrospective Analysis From the Medical Information Mart for Intensive Care IV Database
Source: JMIR Form Res. 2023 Nov 14;7:e44763. doi: 10.2196/44763 (PMC10685278; doi:10.2196/44763)
Supplement: Multimedia Appendix 1 [file formative_v7i1e44763_app1.pdf]

Multimedia Appendix for:

Machine Learning Algorithms Predict Successful Weaning From Mechanical Ventilation Before Intubation: A Retrospective Analysis from the Medical Information Mart for Intensive Care IV Database

Jinchul Kim, Yun Kwan Kim, Hyeyeon Kim, Hyojung Jung, Soonjeong Koh, Yujeong Kim, Dukyong Yoon, Hahn Yi, Hyung-Jun Kim

This is a Multimedia Appendix to a full manuscript published in the J Med Internet Res. For full copyright and citation information see http://dx.doi.org/10.2196/jmir.44763.

Table S1. Checklist of Guidelines for Developing and Reporting Machine Learning Predictive Models in Biomedical Research

Item number	Topic	Checklist item	Page
**Title**			
1	Nature of study	Identify the report as introducing a predictive model	1
**Abstract**			
2	Structured summary	Background<br>Objectives<br>Data sources<br>Performance metrics of the predictive model or models, in both point estimates and confidence intervals<br>Conclusion including the practical value of the developed predictive model or models	1,2
**Introduction**			
3	Rational	Identify the clinical goal<br>Review the current practice and prediction accuracy of any existing models	2
4	Objectives	State the nature of study being predictive modeling, defining the target of prediction<br>Identify how the prediction problem may benefit the clinical goal	2
**Methods**			
5	Describe the setting	Identify the clinical setting for the target predictive model.<br>Identify the modeling context in terms of facility type, size, volume, and duration of available data.	2
6	Define the prediction problem	Define a measurement for the prediction goal (per patient or per hospitalization or per type of outcome).<br>Determine that the study is retrospective or prospective.<br>Identify the problem to be prognostic or diagnostic.<br>Determine the form of the prediction model: (1) classification if the target variable is categorical, (2) regression if the target variable is continuous, (3) survival prediction if the target variable is the time to an event.<br>Translate survival prediction into a regression problem, with the target measured over a temporal	2-3

		window following the time of prediction.<br>Explain practical costs of prediction errors (eg, implications of underdiagnosis or overdiagnosis).<br>Defining quality metrics for prediction models.<br>Define the success criteria for prediction (eg, based on metrics in internal validation or external validation in the context of the clinical problem).	
7	Prepare data for model building	Identify relevant data sources and quote the ethics approval number for data access.<br>State the inclusion and exclusion criteria for data.<br>Describe the time span of data and the sample or cohort size.<br>Define the observational units on which the response variable and predictor variables are defined.<br>Define the predictor variables. Extra caution is needed to prevent information leakage from the response<br>variable to predictor variables.<br>Describe the data preprocessing performed, including data cleaning and transformation. Remove outliers with impossible or extreme responses; state any criteria used for outlier removal.<br>State how missing values were handled.<br>Describe the basic statistics of the dataset, particularly of the response variable. These include the ratio of positive to negative classes for a classification problem and the distribution of the response variable for regression problem.<br>Define the model validation strategies. Internal validation is the minimum requirement; external validation should also be performed whenever possible.<br>Specify the internal validation strategy. Common methods include random split, time-based split, and patient-based split.<br>Define the validation metrics. For regression problems, the normalized root-mean-square error should be used. For classification problems, the metrics should include sensitivity, specificity, positive predictive value, negative predictive value, area under the ROC curve, and calibration plot<br>For retrospective studies, split the data into a derivation set and a validation set. For prospective studies, define the starting time for validation data collection.	3
8	Build the predictive model	Identify independent variables that predominantly take a single value (eg, being zero 99% of the time).<br>Identify and remove redundant independent variables.	3-4

		Identify the independent variables that may suffer from the perfect separation problem.<br>Report the number of independent variables, the number of positive examples, and the number of<br>negative examples.<br>Assess whether sufficient data are available for a good fit of the model. In particular, for classification, there should be a sufficient number of observations in both positive and negative classes.<br>Determine a set of candidate modeling techniques (eg, logistic regression, random forest, or deep learning). If only one type of model was used, justify the decision for using that model.<br>Define the performance metrics to select the best model.<br>Specify the model selection strategy. Common methods include K-fold validation or bootstrap to estimate the lost function on a grid of candidate parameter values. For K-fold validation, proper stratification by the response variable is needed.<br>For model selection, include discussion on (1) balance between model accuracy and model simplicity or interpretability, and (2) the familiarity with the modeling techniques of the end user	
**Results**			
9	Report the final model and performance	Report the predictive performance of the final model in terms of the validation metrics specified in the methods section.<br>If possible, report the parameter estimates in the model and their confidence intervals. When the direct calculation of confidence intervals is not possible, report nonparametric estimates from bootstrap samples.<br>Comparison with other models in the literature should be based on confidence intervals.<br>Interpretation of the final model. If possible, report what variables were shown to be predictive of the response variable. State which subpopulation has the best prediction and which subpopulation is most difficult to predict.	5-6
**Discussion**			
10	Clinical implications	Report the clinical implications derived from the obtained predictive performance. For example, report the dollar amount that could be saved with better prediction. How many patients could benefit from a care model leveraging the model prediction? And to what extent?	8
11	Limitations	Discuss the following potential limitations:	9

	of the model	• Assumed input and output data format<br>• Potential pitfalls in interpreting the model<br>• Potential bias of the data used in modeling<br>• Generalizability of the data	
12	Unexpected results during the experiments	Report unexpected signs of coefficients, indicating collinearity or complex interaction between predictor variables	8-9

Table S2. Additional Detailed Information on Patients in the Intensive Care Unit According to Successful Weaning from Mechanical Ventilation within 14 Days

**Variables**	**Total (n = 23,242)**	**Successful weaning (N = 19,025)**	**Prolonged mechanical ventilation or mortality (n = 4,217)**	***P***
GCS score-eye				< 0.001
No eye opening	13458 (57.9)	11235 (59.1)	2223 (52.7)	
Eyes open to pain	1923 (8.3)	1559 (8.2)	364 (8.6)	
Eyes open to verbal command	2820 (12.1)	2362 (12.4)	458 (10.9)	
Eyes open spontaneously	5041 (21.7)	3869 (20.3)	1172 (27.8)	
GCS score-motor				< 0.001
No motor response	11021 (47.4)	9470 (49.8)	1551 (36.8)	
Decerebrate posture	224 (1.0)	138 (0.7)	86 (2.0)	
Decorticate posture	231 (1.0)	114 (0.6)	117 (2.8)	
Withdraws from pain	2214 (9.5)	1601 (8.4)	613 (14.5)	
Purposeful movement for painful stimulus	3000 (12.9)	2464 (13.0)	536 (12.7)	
Obeys commands	6552 (28.2)	5238 (27.5)	1314 (31.2)	
Admission type				< 0.001
Medical	13625 (58.6)	10418 (54.8)	3207 (76.0)	
Unscheduled surgical	8286 (35.7)	7307 (38.4)	979 (23.2)	
Scheduled surgical	1331 (5.7)	1300 (6.8)	31 (0.7)	
Public insurance	11641 (50.1)	9259 (48.7)	2382 (56.5)	< 0.001
English as the primary language	20951 (90.1)	17186 (90.3)	3765 (89.3)	0.04
Married	10969 (47.2)	9286 (48.8)	1683 (39.9)	< 0.001
Ethnicity				0.009
White	18932 (81.5)	15491 (81.4)	3441 (81.6)	
Black	1759 (7.6)	1399 (7.4)	360 (8.5)	
Hispanic	762 (3.3)	646 (3.4)	116 (2.8)	
Asian	597 (2.6)	491 (2.6)	106 (2.5)	
Others	1192 (5.1)	998 (5.2)	194 (4.6)	
Care unit				< 0.001
Medical	5157 (22.2)	3625 (19.1)	1532 (36.3)	

Surgery	15478 (66.6)	13488 (70.9)	1990 (47.2)	
Others	2607 (11.2)	1912 (10.0)	695 (16.5)	
Underlying comorbidities				
Congestive heart failure	6119 (26.3)	4841 (25.4)	1278 (30.3)	< 0.001
Chronic pulmonary disease	5736 (24.7)	4640 (24.4)	1096 (26.0)	0.030
Diabetes without complication	5454 (23.5)	4463 (23.5)	991 (23.5)	0.952
Myocardial infarction	4536 (19.5)	3708 (19.5)	828 (19.6)	0.830
Chronic kidney disease	4191 (18.0)	3207 (16.9)	984 (23.3)	< 0.001
Cerebrovascular disease	3454 (14.9)	2379 (12.5)	1075 (25.5)	< 0.001
Peripheral vascular disease	3011 (13.0)	2409 (12.7)	602 (14.3)	0.005
Mild liver disease	2810 (12.1)	1944 (10.2)	866 (20.5)	< 0.001
Malignant cancer	2373 (10.2)	1716 (9.0)	657 (15.6)	< 0.001
Diabetes with complication	2068 (8.9)	1690 (8.9)	378 (9.0)	0.858
Severe liver disease	1326 (5.7)	877 (4.6)	449 (10.6)	< 0.001
Paraplegia	1067 (4.6)	716 (3.8)	351 (8.3)	< 0.001
Metastatic solid tumor	1029 (4.4)	679 (3.6)	350 (8.3)	< 0.001
Rheumatic disease	704 (3.0)	564 (3.0)	140 (3.3)	0.233
Peptic ulcer disease	632 (2.7)	495 (2.6)	137 (3.2)	0.021
Dementia	595 (2.6)	424 (2.2)	171 (4.1)	< 0.001
AIDS	109 (0.5)	86 (0.4)	23 (0.5)	0.454

Numbers are presented as counts (percentiles). Abbreviations: GCS, Glasgow Coma Scale; AIDS, acquired immunodeficiency syndrome.

Table S3. Comparison of patients with or without the values for SOFA and SAPS II scores

Variables	Patients with SOFA/SAPS II (n = 23,242)	Patients without SOFA/SAPS II (n = 915)	*P*
Age, years	64.4 ± 15.9	66.0 [56.0 – 77.0]	0.406
Male sex	14,274 (61.4)	530 (57.9)	0.036
Height, cm	170.0 ± 12.9	NA	NA
Weight, kg	83.3 ± 23.4	85.9 ± 25.5	0.005
Body temperature, ℃	36.6 ± 1.4	36.7 ± 1.1	0.028
WBC count, per 10^9/L	13.0 ± 7.6	13.3 ± 7.7	0.394
NL ratio	11.2 ± 14.7	17.1 ± 21.5	<0.001
Hemoglobin, g/dL	10.4 ± 2.16	10.0 ± 2.0	<0.001
Platelet count, per 10^9/L	191.0 ± 102.0	196.0 ± 128.0	0.136
BUN, mg/dL	24.4 ± 19.5	33.5 ± 25.5	<0.001
Creatinine, mg/dL	1.3 ± 1.3	1.6 ± 1.6	<0.001
Albumin, g/dL	3.2 ± 0.8	3.0 ± 0.7	<0.001
Total bilirubin, mg/dL	1.9 ± 4.5	4.5 ± 9.1	<0.001
Prothrombin time, INR	1.5 ± 0.7	1.6 ± 0.8	<0.001
pH	7.3 ± 0.1	7.4 ± 0.1	<0.001
Sodium, mEq/L	139.0 ± 4.7	139.0 ± 5.9	0.024
Potassium, mEq/L	4.2 ± 0.70	4.1 ± 0.6	<0.001
Lactate, mmol/L	2.7 ± 2.1	2.4 ± 2.2	<0.001
Bicarbonate, mEq/L	23.0 ± 4.7	24.2 ± 5.35	<0.001
Anion gap, mEq/L	14.4 ± 4.6	15.1 ± 4.7	<0.001
Duration of MV, days	0.7 [0.2 – 2.7]	2.6 [0.8 – 7.4]	<0.001

Successful weaning within 14 days	19,025 (81.9%)	575 (62.8%)	<0.001

Numbers are presented as mean ± standard deviations, median [interquartile range], or counts (percentiles) as appropriate. Abbreviations: NA, not available; SOFA, Sequential Organ Failure Assessment; SAPS II, Simplified Acute Physiology Score II; WBC, white blood cell; NL, neutrophil/lymphocyte; BUN, blood urea nitrogen; INR, international normalized ratio; MV, mechanical ventilator.

Figure S1. Examples of the patient selection according to keywords in the procedure or chart events.

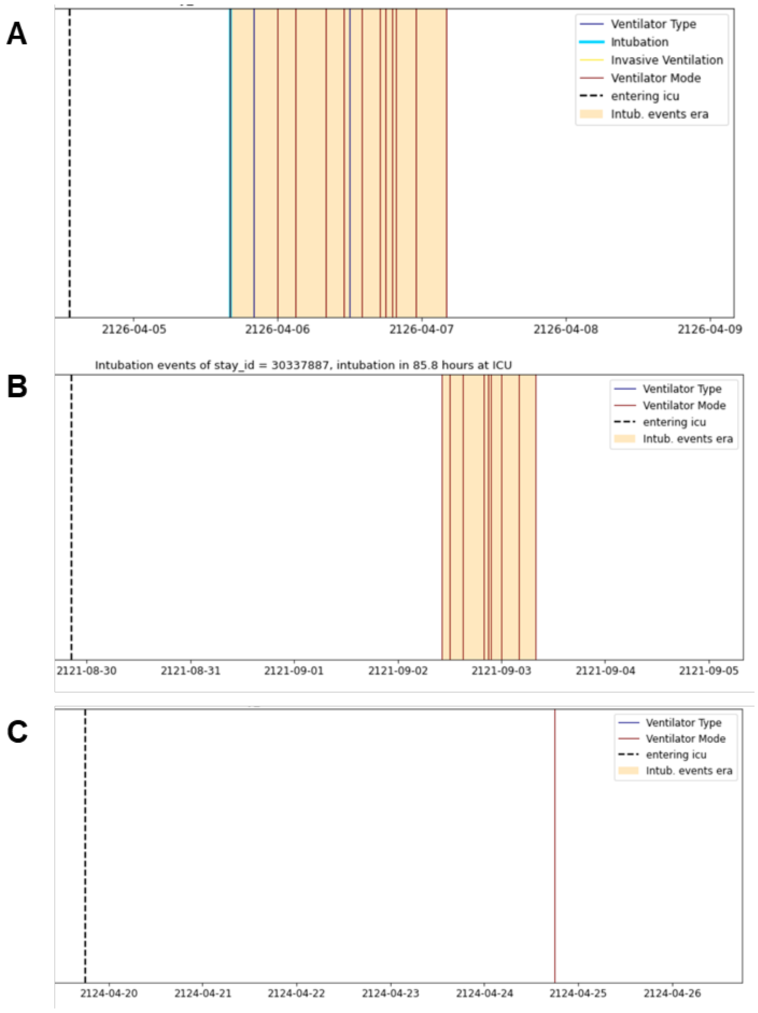

(*A*) An example with “intubation” and “invasive ventilation” appears first, followed by repeated recordings about ventilator modes. (*B*) An example without “intubation” or “invasive ventilation” records but with recordings of ventilator modes ≥ five times within 24 hours. (*C*) An example without “intubation” or “invasive ventilation” recordings and only a single recording of ventilator mode. Patients A and B were considered to have been intubated, whereas patient C was not.

Figure S2. Distributions of all explanatory variables along with SOFA scores and SAPS II.

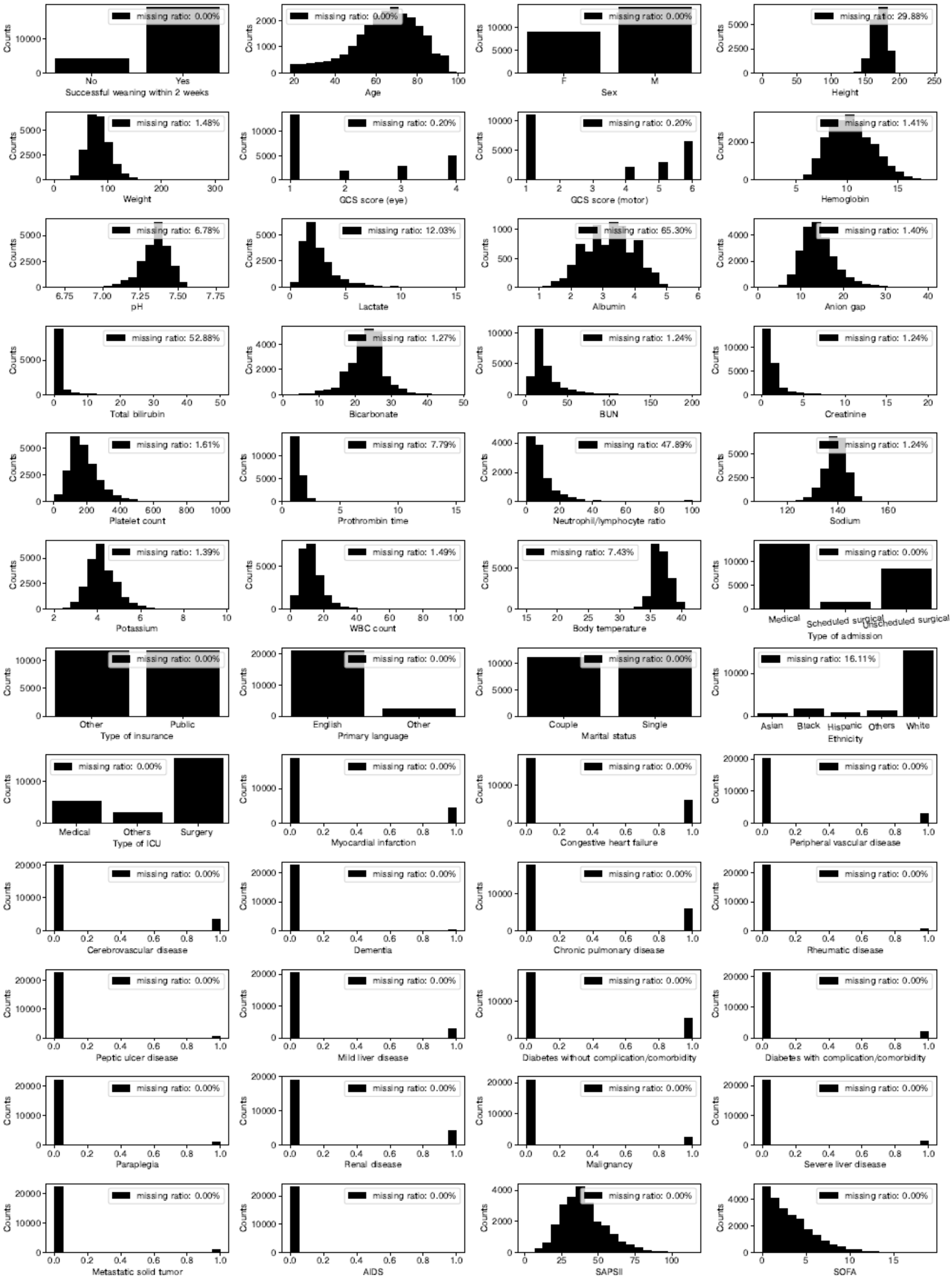

The rates of missing variables are shown in the legend.

Figure S3. Calibration curves of the machine learning algorithms

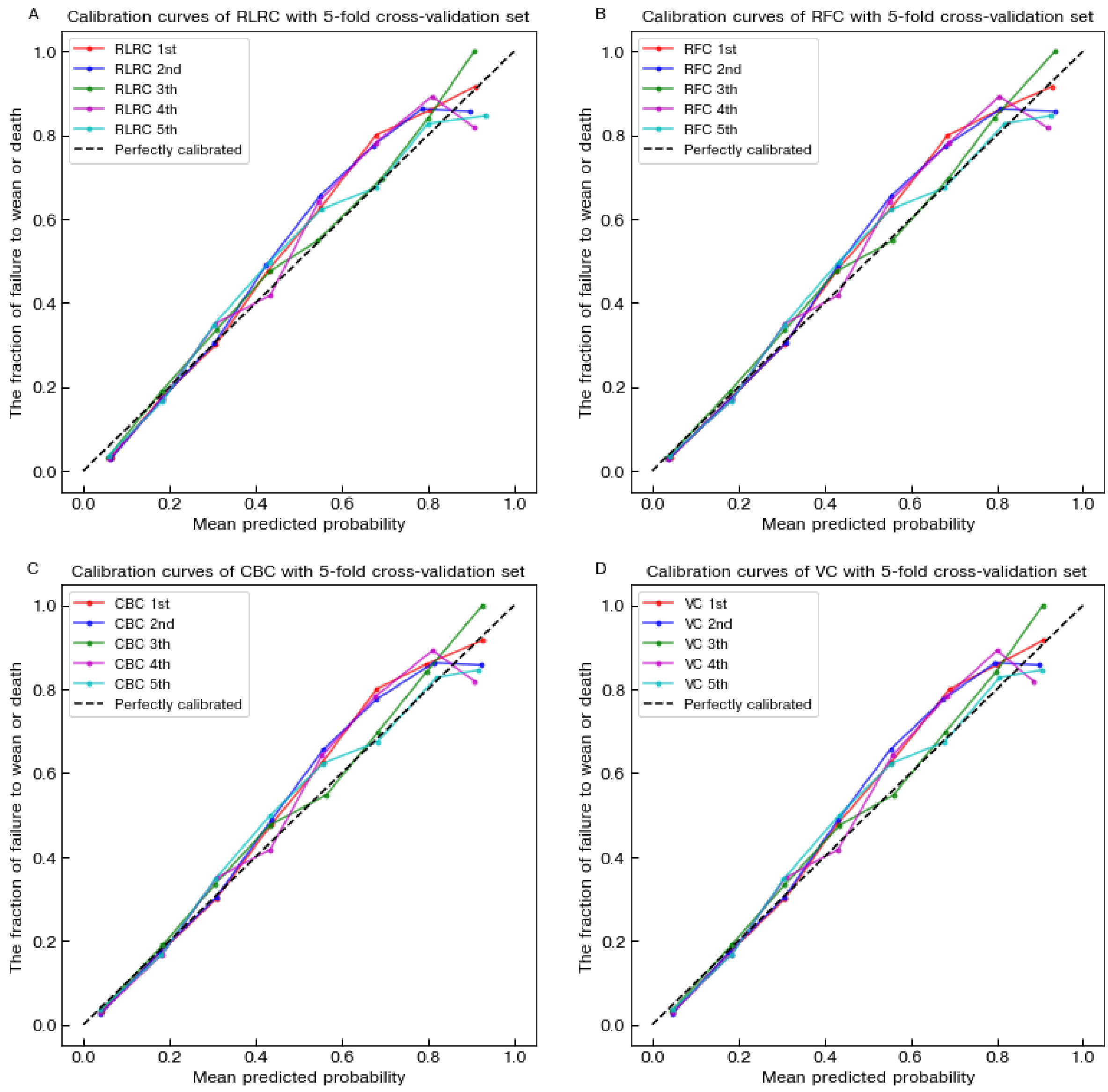

Abbreviations: RLRC, regularized logistic regression classifier; RFC, random forest classifier; CBC, CatBoost classifier; VC, voting classifier.

Figure S4. Confusion matrix of the final ensemble voting classifier model.

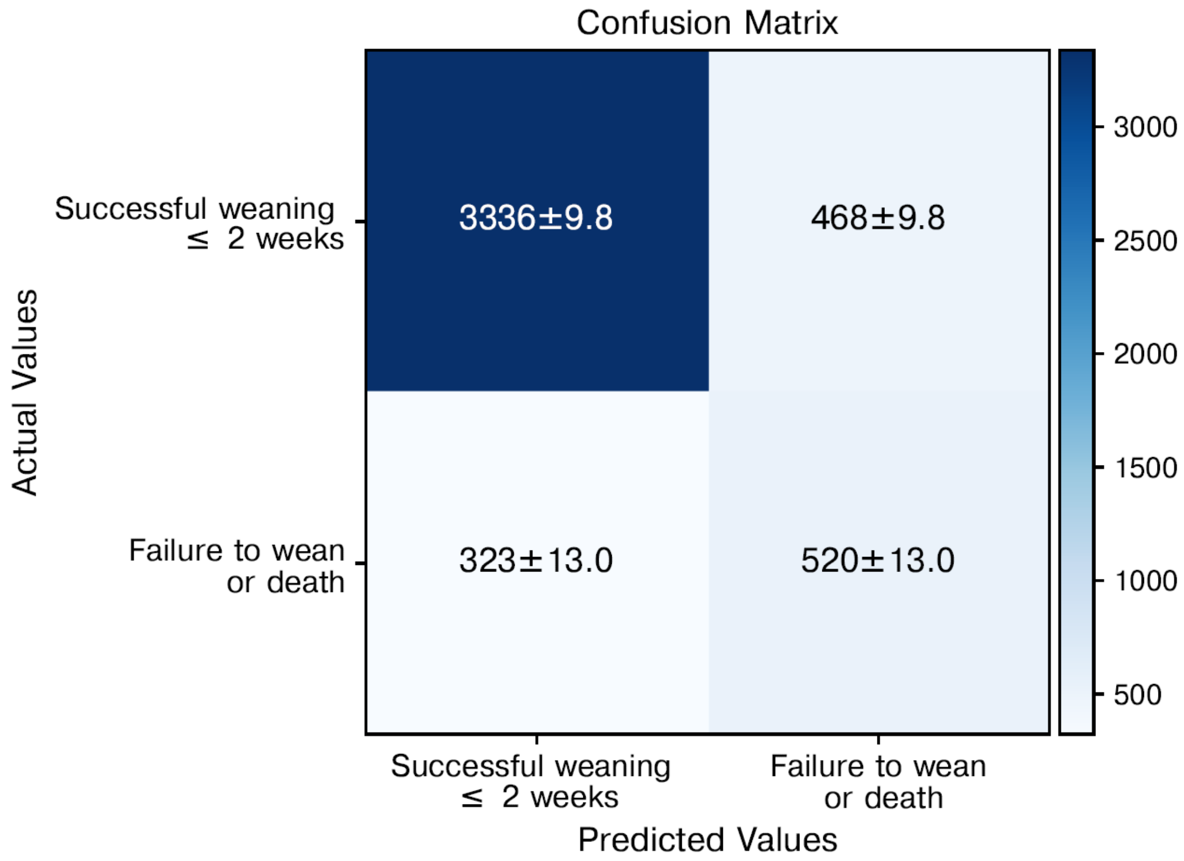

The confusion matrix was computed using Cohen's kappa, maximizing the threshold value. The maximum value of Cohen's kappa was 0.456, with a threshold of 0.31. The numbers in the confusion matrix cells are the mean and standard deviation, calculated using a 5-fold cross-validation set. The upper left cell represents a true negative, the upper right cell a false positive, the lower left cell a false negative, and the lower right cell a true positive.
